# Mixed Impact of Firearms Restrictions on Fatal Firearm Injuries in Males: A National Observational Study

**DOI:** 10.3390/ijerph110100487

**Published:** 2013-12-30

**Authors:** Finn Gjertsen, Antoon Leenaars, Margarete E. Vollrath

**Affiliations:** 1 Department of Psychosomatics and Health Behavior, Norwegian Institute of Public Health, P.O. Box 4404 Nydalen, 0403 Oslo, Norway; E-Mail: MargareteErika.Vollrath@fhi.no; 2 Psychologist in Private Practice, 1500 Ouellette Avenue, Windsor, ON, N8X 1K7, Canada; E-Mail: draalee@sympatico.ca; 3 Department of Psychology, University of Oslo, P.O. Box 1094 Blindern, 0317 Oslo, Norway

**Keywords:** accident, firearm deaths, firearms legislation, gun control, means restriction, military, suicide, unintentional firearm deaths

## Abstract

*Introduction*: Public health organizations have recommended restricted access and safe storage practices as means to reduce firearm injuries and deaths. We aimed to assess the effect of four firearm restrictions on firearm deaths in Norway 1969–2009. *Methods*: All deaths due to firearm discharge were included (5,660 deaths, both sexes). The statistical analysis to assess impact of firearm legislations was restricted to males because of the sex disproportionality (94% were males). *Results*: A total of 89% of firearm deaths (both sexes) were classified as suicide, 8% as homicide, and 3% as unintentional (accident). During the past four decades, male accidental firearm death rates were reduced significantly by 90%. Male firearms suicide rates increased from 1969 to 1991 by 166%, and decreased by 62% from 1991 to 2009. Despite the great reduction in male accidental firearm deaths, we were unable to demonstrate effects of the laws. In contrast, we found that a 1990 regulation, requiring a police permit before acquiring a shotgun, had a beneficial impact on suicide in the total sample and in those aged 15–34 years. Male firearm homicides decreased post-2003 regulation regarding storing home guard weapons in private homes. *Conclusions*: Our findings suggest that two laws could have contributed to reduce male firearm mortality. It is, however, a challenge to measure the role of four firearm restrictions. The null findings are inconclusive, as they may reflect no true impact or study limitations.

## 1. Introduction

Injuries and deaths resulting from the discharge of a firearm are a public health concern in many countries worldwide. In Europe and other high-income countries, suicide is the most frequent cause of firearm mortality, followed by homicide and unintentional (accidental) fatalities [1]. Unintentional firearm deaths comprise a small but significant fraction of all firearm deaths [2], as injury from firearm discharge has high case fatality [3,4].

Restricted access and safe storage practices have been recommended by public health organizations, such as the World Health Organization, the American Medical Association, and the Centers for Disease Control and Prevention in the United States, as means to reduce the incidence of firearm injuries and deaths [4,5,6,7,8]. Several studies provide empirical support for the hypothesis that high availability of firearms is associated with an increased risk of fatal firearm injuries, especially homicides and suicides [4,9,10]. Independent of firearm prevalence, the greatest risk for unintentional firearm deaths in the United States was found in states where gun owners were more likely to store their firearms loaded and unlocked [7].

More studies have supported the notion that firearm regulations are followed by a significantly reduced incidence of suicides [11,12,13], homicides [4,14], and unintentional deaths [15] caused by firearms. For suicide, restricted firearm access has reportedly had the greatest impact among young males [4,11]. However that may be, the effectiveness of stringent firearms legislation as an evidence-based public health strategy to reduce firearm-related deaths is debated, and available scientific evidence is not conclusive. This issue is exemplified by studies that report inconsistent results for suicide deaths when assessing the impact of the same laws: Bill C-17, secure safe storage, in Canada [3,4,7,11,16], and the 1996 gun law reforms that resulted in the removal of approximately 650,000 semiautomatic rifles, shotguns, and pump-action shotguns from private owners in Australia [17,18,19]. Hahn *et al*. [20] provided a comprehensive systematic review of firearms laws in the United States and studies of their effects, concluding that the evidence is insufficient to determine whether firearms laws have affected intentional (suicide and homicide) or unintentional fatal firearm injuries. More recently, other studies have reported uplifting results regarding the impact of firearm reform legislation on the rates of firearm homicides and suicides in Austria [21], firearm suicide in the United States [13], and firearm suicides among young males in Canada [11], as well as on the effect of a mandatory hunter’s exam on unintentional firearm mortality in Sweden [15].

Norway has a relatively high prevalence of firearms; in 2008, the Norwegian police registered approximately 1.4 million private firearms owned by approximately 500,000 individuals (Marte Ø. Lund, Directorate of Police, personal communication 21 June 2011). This data provides a rough estimate that 24% of households have firearms in a population of 4.8 million individuals (~2.1 million private households). Whether the prevalence of firearms has changed during the last few decades is unknown. One study estimated that 32% of households in Norway owned firearms in 1989 [22]; however, the authors did not report the data source. During the last four decades, Norwegian gun control laws have gradually become more restrictive, affecting legal sales and the inheritance of firearms, establishing a national firearm registry, regulating the storage of privately owned firearms, instituting a mandatory exam for hunters, and removing military weapons stored in private homes. The laws are as follows:

1. Hunter’s examination (1986): Since 1 April 1986, it has been obligatory for new hunters to pass a 30 h course with theoretical and practical training (changes to the Act of Game of 1981) [23]. Individuals registered as hunters before that date did not have to pass the examination; however, all hunters born after 31 March 1970 have passed.
2. Shotgun acquisition (1990): Since 1 October 1990, the acquisition of any type of shotgun has required permission from the police, like the acquisition of other firearms (changes to the Act of Firearms and Explosives of 1961). Individuals who want to acquire a firearm must provide a reason, and the firearm will be registered in the national firearm registry.
3. Weapons cabinet (2000): Since 1 September 2000, it has been obligatory for households with five or more rifles and/or shotguns, or one or more pistols, revolvers, or semi-automatic and automatic firearms, to store private firearms in a locked weapons cabinet (changes to the Act of Firearms and Explosives of 1961). Since 1 July 2010, this regulation has been enforced from the first rifle/shotgun, but this new change to the law was not taken into consideration in the present study.
4. Home Guard firearm (2003): Beginning 1 January 2003, the Norwegian Armed Forces ordered removal of the percussion cap of any Home Guard weapon stored in a private home (changes to the Act of the Home Guard of 1953).

Generally speaking, we assume that these four firearm regulations caused changes to the three dependent variables “fatal firearm accident”, “firearm homicide”, and “firearm suicide”. Potential links to the dependent variables might involve intermediates, such as attitudes and behaviors related to the handling of firearms for hunting and sport, or a more direct impact, e.g., restricting access to firearms might force people to abandon suicide plans when the method is unavailable, rather than switch to another method [24,25], especially for young suicides [11,24]. Including shotguns on the list of weapons for which police permission is needed before acquisition may prevent suicides, especially in cases where the suicidal crisis and plans are short-lived and influenced by impulsivity and alcohol [3,24], and safer storage might result in fewer fatal firearm accidents in homes with guns [7].

The aim of the present study was to assess the impact of four firearm laws on trends in fatal firearm injuries over four decades in the Norwegian population. More specifically, we expected to find a reduced risk after the introduction of three of the four laws: (1) The mandatory training of hunters with a focus on safe use, transport, and storage of firearms may potentially reduce the risk of unintentional firearm deaths related to hunting and at home; (2) Restricting the acquisition of shotguns may lower the risk of firearm suicides among persons who do not own or have access to a weapon; (3) Removal of the percussion cap of military weapons stored in private homes may reduce the risk of homicides and suicides. In contrast, we did not expect the legislative amendment that introduced storage of firearms in a weapon cabinet if ≥5 long guns (or ≥1 handguns) in a household to affect the trends of firearm death rates. Even if this law worked well to reduce injury episodes, it only affected owners of five or more long guns, or handgun(s). Regarding firearm type, we assessed only the manner of intent (*i.e.*, accident, homicide, or suicide) because detailed data on type of firearm was only available after 1996.

## 2. Materials and Methods

We collected national mortality data from the Norwegian Institute of Public Health. The data covered a period of 41 years, from 1969 to 2009. Three revisions of the International Classification of Diseases (ICD) were used to code the cause of death: Norwegian editions of ICD-8 and ICD-9, and the English version of ICD-10 [26] (Table 1). The cause-specific mortality registry was compiled by Statistics Norway. The mortality registry includes persons registered as residents in Norway at the time of death, regardless of whether the death occurred in Norway or abroad [27].

**Table 1:** Matrix for firearm mortalities.

Manner of intent (external causes of death)	ICD-10	ICD-9 ^a^	ICD-8 ^a^
**Unintentional, Accidental**	**W32-W34**	**E922** ^b^	**E922**
Handgun	W32		
Rifle, shotgun, and larger firearm	W33		
Other and unspecified	W34		
**Intentional, Suicide**	**X72-X75**	**E955** ^c^	**E955**
Handgun	X72		
Rifle, shotgun, and larger firearm	X73		
Other and unspecified	X74		
Explosive material	X75		
**Intentional, Homicide**	**X93-X96**	**E965** ^c^	**E965**
Handgun	X93		
Rifle, shotgun, and larger firearm	X94		
Other and unspecified	X95		
Explosive material	X96		
**Event of undetermined intent**	**Y22-Y25**	**E985** ^c^	**E985**
Handgun	Y22		
Rifle, shotgun, and larger firearm	Y23		
Other and unspecified	Y24		
Explosive material	Y25		
**Other intent**	**Y35.0, Y36.4**	**E970, E991**	**E970, E991**
Legal invention involving firearm	Y35.0	E970	E970
Operation of war involving firearm	Y36.4	E991	E991

^a^ The Norwegian edit of the classification was used for mortality statistics; ^b^ Fifth-character subdivisions of the code were not used; ^c^ Fifth-character subdivisions were not used for this code, and the four-character code includes both firearm discharge and explosive material.

We included all deaths classified as resulting from the discharge of a firearm as the underlying cause of death. Because firearm deaths were infrequent among females, the analysis was limited to males. To create comparable categories across ICD-8, ICD-9, and ICD-10, deaths caused by explosive materials were included in three categories: homicide, suicide, and event of undetermined intent (Table 1).

Population figures were collected from Statistics Norway, and we used information about changes to civil and military acts and legislation related to firearms.

### 2.1. Classification of the Type of Firearm

ICD-8 does not contain codes for the type of firearm [28]. The Norwegian edition of ICD-9 had fifth-digit subdivisions specifying the type of firearm in four codes: E922 (accident caused by firearm missile), E955 (suicide by firearms and explosives), E965 (assault by firearms and explosives), and E985 (injury by firearm and explosives, undetermined whether accidentally or purposefully inflicted) [29]. The five firearm categories in ICD-9 were: *Hand gun* (including any gun used with one hand, pistols, and revolvers); *Shotgun* (automated); *Hunting rifle*; *Military firearms* (army gun); and *Other and unspecified firearms*. Because shotguns and hunting and military firearms were blended into a single category in ICD-10, substantial information about the specificity of firearm type was lost from ICD-10 compared with ICD-9 [30]. In ICD-9, air guns were shifted from the code for accidental firearm injuries (E922) to the code E917 (striking against or struck accidentally by objects). In ICD-10, air gun accidents are included in code W34 (discharge from other and unspecified firearms).

However, the subdivisions in ICD-9 that specified firearm type were not used in the Norwegian mortality statistics. Therefore, the only information available about the type of firearms in this study was from 1996, when ICD-10 was implemented.

### 2.2. Reference Data

Any significant change in the trends of firearm deaths after the introduction of firearms legislation is difficult to interpret without making comparisons with trends in non-firearm injury deaths. We used reference data (controls) for suicide and homicide: non-firearm suicide and non-firearm homicide, respectively. For unintentional incidents (accidents), it is more complicated to theorize how a change in unintentional firearm deaths should be associated with changes in other unintentional injury mechanisms (e.g., drowning, poisoning, motor vehicle traffic, and falls). Therefore, changes in accidents due to mechanisms other than firearms should probably not affect any association between firearm accidents and firearms legislation.

The annual number of deaths in the three male firearms injury categories and the two reference categories are presented in Table 2. The regression analysis was confined to males, as 94% of all firearm deaths occurred in males (Table 3).

**Table 2:** Male deaths divided into three firearm injury categories and two non-firearm injury categories. These data were used in the Poisson analysis.

	**Firearm**			**Non-firearm**	
	1. Accidental	2. Suicide	3. Homicide	1. Suicide	2. Homicide
**Total 1969–2009**	**158**	**4,863**	**276**	**10,756**	**767**
1969	8	73	1	177	19
1970	7	79	4	149	13
1971	9	54	3	184	13
1972	4	73	5	181	13
1973	10	64	6	194	14
1974	11	102	3	220	10
1975	3	90	6	193	12
1976	7	96	3	223	14
1977	6	132	5	208	20
1978	4	122	3	228	17
1979	3	110	7	238	19
1980	7	121	4	249	22
1981	3	132	13	256	24
1982	10	148	5	273	19
1983	6	158	7	275	24
1984	5	158	8	290	27
1985	1	142	5	286	20
1986	3	159	12	260	33
1987	2	178	4	308	27
1988	6	199	15	311	24
1989	1	185	7	296	25
1990	4	157	12	331	25
1991	7	193	15	306	31
1992	3	161	8	288	26
1993	5	164	7	285	16
1994	3	118	7	261	11
1995	5	136	6	275	22
1996	4	128	7	261	18
1997	2	126	5	261	23
1998	2	98	9	301	20
1999	1	116	10	314	14
2000	-	107	11	302	18
2001	-	100	4	311	16
2002	-	87	10	275	16
2003	-	101	11	273	14
2004	-	90	10	269	15
2005	2	79	3	281	14
2006	1	88	7	303	13
2007	2	60	2	276	15
2008	-	83	2	264	17
2009	1	96	4	320	14

Nile = -.

**Table 3:** Summary of fatal firearm injuries 1969–2009 by manner of intent.

Manner of intent ^a,b^	Total N (%)	Mean	Std. Dev.	Min	Max
**Total firearm deaths**	**5,660 (100)**	**138.1**	**43.5**	**67**	**243**
Accidental	164 (2.9)	4	3.2	-	11
Suicide ^a^	5,025 (88.8)	122.6	40	57	212
Homicide ^a^	432 (7.6)	10.5	4.7	2	24
Event of undetermined intent ^a^	34 (0.6)	0.8	0.9	-	3
Legal investigation.	- (-)	-	-	-	-
Operation of war ^c^	5 (0.1)	0.1	0.3	-	1
**MALE**					
**Total firearm deaths**	**5,334 (100)**	**130.1**	**40.2**	**64**	**221**
Accidental	158 (3.0)	3.9	3.1	-	11
Suicide ^a^	4,863 (91.2)	118.6	37.9	54	199
Homicide ^a^	276 (5,2)	6.7	3.6	1	15
Event of undetermined intent ^a^	32 (0.6)	0.8	0.9	-	3
Legal investigation	- (-)	-	-	-	-
Operation of war ^c^	5 (0.1)	0.1	0.3	-	1
**FEMALE**					
**Total firearm deaths**	**326 (100)**	**8**	**4.1**	**1**	**22**
Accidental	6 (1.8)	0.2	0.4	-	1
Suicide ^a^	162 (49.7)	4	2.7	-	13
Homicide ^a^	156 (47.9)	3.8	2.3	-	10
Event of undetermined intent ^a^	2 (0.6)	0.1	0.2	-	1
Legal investigation	- (-)	-	-	-	-
Operation of war ^c^	- (-)	-	-	-	-

^a^ To make the categories compatible across the 8th, 9th, and 10th Revisions of the International Statistical Classification of Diseases (ICD), deaths by explosive materials are included in the categories for suicide, homicide, and event of undetermined intent; ^b^ See Table 1 for category definitions; ^c^ Bullets and fragments.

### 2.3. Piecewise Regression

We tested the impact of legal regulations on fatal firearm injuries to males using a piecewise regression model [31]. The study period 1969–2009 was subdivided into five time periods defined by the years in which the four laws were implemented. We used Poisson regression to test whether the trend changed significantly between two successive periods (*i.e.*, if the regression coefficient represented by the slope of the line changed from the previous period) [31]. The slope in the first period was tested to determine whether it differed significantly from zero. Negative binomial regression is considered more suitable for count data than Poisson regression owing to its capacity to account for over-dispersion [19]. However, these two regression models do not differ substantially; we observed no differences when we compared the two regression models.

**Figure 1:**
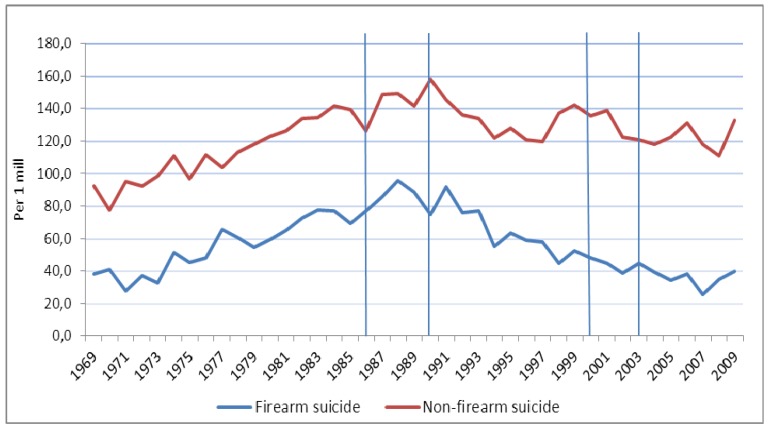
Observed time trends in male firearm suicide and male non-firearm suicide in Norway 1969–2009 (rates per 1 million). Different pieces of firearms legislation were implemented in the years 1986, 1990, 2000, and 2003 (vertical lines).

Poisson regression was also used to estimate the annual change (rate ratio) in unintentional and intentional firearm mortality rates and in non-firearm suicide and non-firearm homicide, with the mean population as the exposure variable and the year as a covariate (independent variable). Because firearm and non-firearm suicide and homicide death rates exhibited nonlinearity during the period 1969–2009 (Figure 1 and Figure 3), we ran separate regressions for each of these four categories. For example, for non-firearm suicide we ran one analysis from 1969 to 1990, another from 1990 to 1994, and a third from 1994 to 2009 (Table 4). We analyzed the data using the count-data function in the statistical software Stata/SE version 12.1 [32].

**Table 4:** Annual estimated change in three categories of male firearm death rates, and the reference categories (non-firearm suicide rates and non-firearm homicide rates) using Poisson regression.Firearm and non-firearm suicide and homicide exhibited nonlinearity during the period of 1969–2009, and separate regressions for each of these categories are presented.

Manner of intent	Rate Ratio	Std. Error	*p*	95% CI	
**Firearm accidental**					
1969–2009	0.9434566	0.0070837	<**0.001**	0.9296744	0.9574431
**Firearm homicide**					
1969–1988	1.063483	0.0176508	<**0.001**	1.029445	1.098647
1988–2009	0.9523446	0.0116701	<**0.001**	0.9297441	0.9754944
**Non-firearm homicide**					
1969–1986	1.045128	0.0112655	<**0.001**	1.02328	1.067443
1986–2009	0.9601498	0.0065133	<**0.001**	0.9474685	0.9730009
**Firearm suicide**					
1969–1988	1.052911	0.0038548	<**0.001**	1.045383	1.060493
1988–2009	0.9483321	0.0029616	<**0.001**	0.9425451	0.9541546
**Non-firearm suicide**					
1969–1990	1.027825	0.002248	<**0.001**	1.023428	1.03224
1990–1994	0.9413462	0.0173876	**0.001**	0.9078767	0.9760497
1994–2009	0.9968206	0.0032004	0.321	0.9905677	1.003113

Although descriptive statistics are presented for both males and females, the trends for the outcome variables and the statistical tests of the effect of legal regulations were confined to males. Results of the Poisson analysis were placed in parentheses if there were fewer than 20 deaths in a period.

### 2.4. Ethics

No governmental or ethical approval was needed because we only used aggregated tabular data at the national level [26].

## 3. Results

A total of 5,660 firearm deaths (5,334 males and 326 females) were registered during the 41-year study period. Approximately 89% of all firearm deaths were classified as suicide; 8% were classified as homicide, 3% were classified as unintentional (accident), and <1% were classified as an event of undetermined intent or as an operation of war (Table 3). Ninety-one percent of firearm deaths among males were suicide, while 50% of firearm deaths among females were suicide. Homicide was the underlying cause of 48% of all female firearm deaths. Deaths caused by unintentional firearm discharge (accident) were rare. In total, 164 accidental firearm deaths were registered during the study period, and nearly all were males (N = 158). Fifteen percent of the accidental firearm deaths occurred in children younger than 15 years of age.

### 3.1. Time Trends in Males

Trends for unintentional (accidental) and intentional (homicide and suicide) firearm deaths among males varied throughout the study period. Male firearm suicide rates exhibited nonlinearity, with an inverse “U”-shaped trend that reached a maximum in 1988. Non-firearm suicide rates also exhibited nonlinearity, reaching a maximum in 1990 (Figure 1). The estimated male suicide rate increased by 166.4% (95% CI 132.4–205.2; *p* < 0.001) from 1969 to 1988, and decreased by 67.2% (95% CI 71.1–62.7; *p* < 0.001) from 1988 to 2009. Male non-firearm suicides increased significantly by 80.0% (95% CI 62.6–94.7; *p* < 0.001) from 1969 to 1990 and decreased by 21.5% (95% CI 32.1–13.5; *p* = 0.001) from 1990 to 1994 followed by stable rates (non-significant changes) from 1994 to 2009 (Table 4).

**Figure 2:**
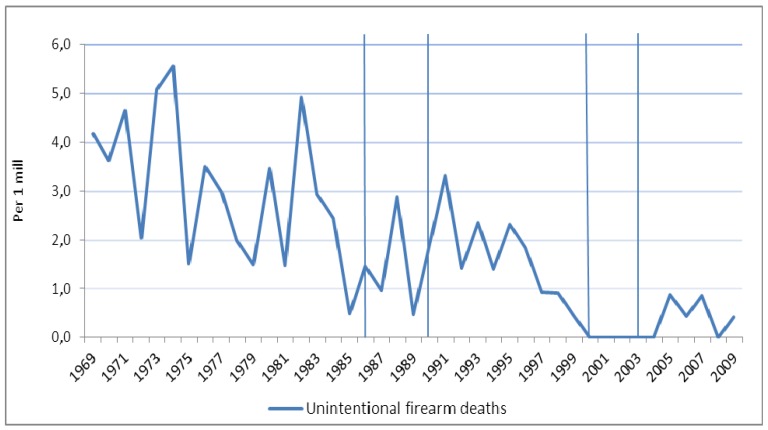
Observed time trend in unintentional (accidental) firearm deaths among males in Norway 1969–2009 (per 1 million). Different pieces of firearms legislation were implemented in 1986, 1990, 2000, and 2003 (vertical lines).

**Figure 3:**
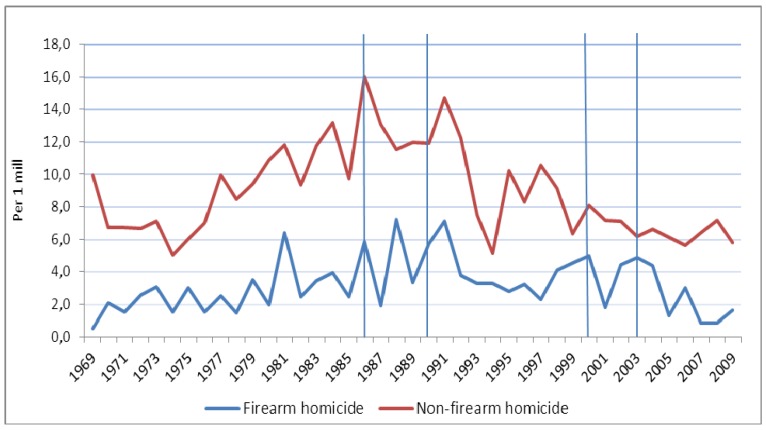
Observed time trends in firearm homicide and non-firearm homicide among males in Norway 1969–2009 (per 1 million). Different pieces of firearms legislation were implemented in 1986, 1990, 2000, and 2003 (vertical lines).

Figure 2 shows the time trends in male unintentional firearm deaths. The estimated trend in unintentional firearm mortality among males decreased significantly by 90.3% (95% CI 94.6–82.4; *p* < 0.001) during the entire period from 1969 to 2009 (Table 4). Figure 3 shows observed trends among males in firearm homicide and non-firearm homicide. The estimated trend in firearm homicides increased significantly by 222.0% (95% CI 73.6–497.5; *p* < 0.001) from 1969 to 1988, and decreased by 64.1% (95% CI 78.3–40.6; *p* < 0.001) from 1988 to 2009. Non-firearm homicides showed a similar pattern; they increased by 111.8% (95% CI 47.9–203.3; *p* < 0.001) from 1969 to 1986, and decreased by 60.8% (95% CI 71.1–46.7; *p* < 0.001) from 1986 to 2009 (Table 4).

### 3.2. Firearms Regulations and Fatality Trends in Males

**Figure 4:**
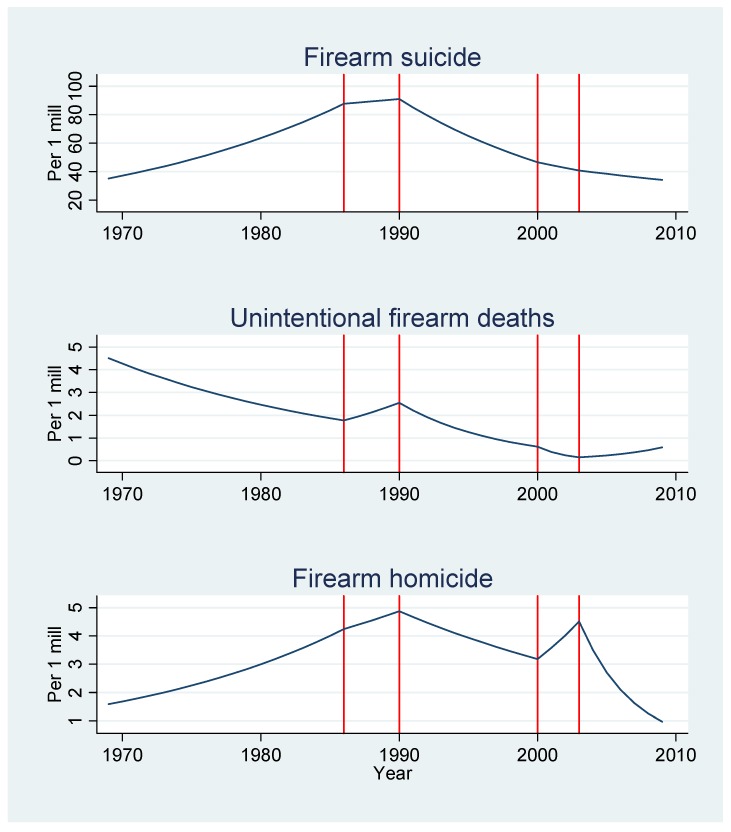
Estimated rates of male firearm deaths (suicide, accidents, and homicide) before and after the four pieces of firearms legislation were introduced (1986, 1990, 2000, and 2003). The estimated trends are per 1 million using a piecewise Poisson regression model.

Table 5 and Figure 4 show the results for the stepwise regression model. The Poisson model indicated that the four pieces of firearms legislation had no effect on unintentional firearm deaths among males. However, the data suggested that a 1990 regulation, requiring a police permit before acquiring a shotgun, could have a beneficial impact on male suicide in the total sample and in the age group 15–34 years old (such beneficial impact was not observed on non-firearm suicide ages 15–34 years as the rates increased significantly post-1990). The model suggested a significant change in male firearm homicides after a 2003 regulation regarding home guard weapons stored in private homes. For male firearm suicides in ages 15–35, the model suggested changes in the rates after introducing a 1986 introduction of mandatory training of hunters with a focus on safety. However, non-firearm suicide rates in the same age groups also decreased significantly post-2003.

The change during the period from 1969 to 1985 was significant in all firearms and non-firearms categories, except non-firearms-related suicide among males aged 65 years and older (Table 5).

**Table 5:** Results of piecewise Poisson regression of the difference in trends of death rates among males during periods defined by four separate firearm regulations ^a^.

Manner of intent ^a^ Period ^b^	Coefficient	95% CI		*p*
**Firearm unintentional deaths (accidents), total (N = 158)**				
1969–1985	−0.0550193	−0.0907301	−0.0193085	**0.003**
1986–1989	0.1461056	−0.0412672	0.3334784	0.126
1990–1999	−0.2324301	−0.472747	0.0078867	0.058
2000–2002 ^b^	(−0.3371303)	(−1.037047)	(0.3627868)	(0.345)
2003–2009 ^b^	(0.7081521)	(−0.2968702)	(1.713174)	(0.167)
**Firearm homicide, total (N = 276)**				
1969–1985	0.0578923	0.0208273	0.0949572	**0.002**
1986–1989	−0.0232726	−0.1657028	0.1191575	0.749
1990–1999	−0.0772099	0.2358867	0.0814669	0.340
2000–2002	0.1589736	0.0784515	0 .3963986	0.189
2003–2009	−0.3724417	0.6899734	0.0549099	**0.022**
**Non-firearm homicide, total (N = 767)**				
1969–1985	0.0437596	0.0237466	0.0637726	**<0.001**
1986–1989	−0.0671237	0.150988	0.0167407	0.117
1990–1999	−0.0239643	−0.122405	0.0744764	0.633
2000–2002	−0.0007202	−0.1646331	0.1631928	0.993
2003–2009	0.0442818	−0.1660476	0.2546112	0.680
**Firearm suicide, total (N = 4,863)**				
1969–1985	0.0527137	0.0445683	0.060859	**<0.001**
1986–1989	−0.0454937	−0.0781764	−0.0128109	**0.006**
1990–1999	−0.0717356	−0.1095559	−0.0339152	**<0.001**
2000–2002	0.0171065	−0.048344	0.082557	0.608
2003–2009	0.0206193	−0.0649099	0.1061486	0.637
**Non-firearm suicide, total (N = 10,756)**				
1969–1985	0.0299268	0.0242818	0.0355718	**<0.001**
1986–1989	−0.0359562	−0.0599087	−0.0120037	**0.003**
1990–1999	−0.0023273	−0.0297866	0.0251319	0.868
2000–2002	−0.0139944	−0.0535758	0.025587	0.488
2003–2009	0.0250989	−0.023887	0.0740847	0.315
**Firearm suicide ages 15–34 years (n = 1,735)**				
1969–1985	0.0809667	0.0667928	0.0951406	**<0.001**
1986–989	−0.081786	−0.1339045	−0.0296674	**0.002**
1990–1999	−0.0781945	−0.1381938	−0.0181952	**0.011**
2000–2002	−0.0676003	−0.1895144	0.0543137	0.277
2003–2009	0.1145374	−0.0556891	0.2847638	0.187
**Non-firearm suicide ages 15–34 years (n = 3,494)**				
1969–1985	0.0513084	0.0404997	0.0621171	**<0.001**
1986–1989	−0.0825061	−0.1252112	−0.0398011	**<0.001**
1990–1999	0.0566463	0.0087228	0.1045699	**0.021**
2000–2002	−0.0410032	−0.1064668	0.0244605	0.220
2003–2009	−0.0085112	−0.0899689	0.0729464	0.838
**Firearm suicide ages 35–64 years (n = 2,250)**				
1969–1985	0.0267295	0.0154617	0.0379973	**<0.001**
1986–1989	−0.0392455	−0.0884941	0.010003	0.118
1990–1999	−0.0478289	−0.1060463	0.0103885	0.107
2000–2002	0.0276482	−0.0686607	0.1239571	0.574
2003–2009	0.0110557	−0.1117119	0.1338234	0.860
**Non-firearm suicide ages 35–54 years (n = 5,336)**				
1969–1985	0.0179062	0.0102051	0.0256072	**<0.001**
1986–1989	−0.0364527	−0.0706378	−0.0022675	**0.037**
1990–1999	−0.003869	−0.0437166	0.0359786	0.849
2000–2002	0.0151949	−0.0423643	0.0727542	0.605
2003–2009	0.0215505	−0.0483767	0.0914776	0.546
**Firearm suicide ages 65+ (n = 850)**				
1969–1985	0.0504112	0.0282675	0.0725548	**<0.001**
1986–1989	−0.009857	−0.0932206	0.0735066	0.817
1990–1999	−0.0725788	−0.1644605	0.0193029	0.122
2000–2002	0.0555168	−0.0819672	0.1930007	0.429
2003–2009	−0.0589224	−0.2313264	0.1134816	0.503
**Non-firearm suicide ages 65+ (n = 1,836)**				
1969–1985	0.0084429	−0.0047017	0.0215875	0.208
1986–1989	0.0308715	−0.0250377	0.0867808	0.279
1990–1999	−0.066876	−0.1307883	−0.0029636	**0.040**
2000–2002	−0.0571535	−0.1592739	0.0449668	0.273
2003–2009	0.0967007	−0.0349453	0.2283467	0.150

^a^ The regression includes males during 1969–2009. Dependent variables are firearm accidents (unintentional), firearm homicides, firearm suicides, non-firearm homicides, and non-firearm suicides. To render the categories compatible across the 8th, 9th, and 10th Revisions of the International Statistical Classification of Diseases (ICD), deaths by explosive materials are included in the categories for homicide and suicide. *p* < 0.05 are in bold type. The laws were implemented in 1986, 1990, 2000, and 2003; ^b^ Regression results based on fewer than 20 deaths during a period are in parentheses.

### 3.3. Firearm Types

Handgun discharges were responsible for 13% of all firearm deaths (in both males and females; Table 6). Discharges from rifles, shotguns, and larger firearms resulting in 43% of deaths; however, a large fraction (45%) were classified as “other and unknown firearms”.

**Table 6:** Deaths due to firearm discharge by type of firearm and intent 1996–2009, % (N).

Type of firearm ICD-10 categories ^a^	Firearm discharge by external cause (intent)				
	Total	Accidental	Suicide	Homicide	Undetermined intent
**Total 100% (N)**	**100 (1,513)**	**100 (17)**	**100 (1,365)**	**100 (127)**	**(4)**
Handgun discharge	12.9 (195)	(3)	11.6 (158)	25.2 (32)	(2)
Rifle, shotgun, and larger firearm	42.5 (643)	(8)	43.4 (592)	33.1 (42)	(1)
Other and unspecified firearms	44.6 (675)	(6)	45.1 (615)	41.7 (53)	(1)

^a^ Data was available from 1996, *i.e.*, the year ICD-10 was implemented on Norwegian mortality statistics.

## 4. Discussion

We observed different patterns of male unintentional and intentional firearm deaths during the four decades from 1969 to 2009, with a great reduction in risk levels from the end of the 1980s, especially of firearm suicide. In fact, male accidental firearm death rates declined during the entire period to a level between one and null per 1 million. We tested whether these encouraging observations might be due to stricter firearms legislation. Our findings did not provide much statistical support to this hypothesis. Given that data about firearm type were not available until 1996 and later—and that those data did not differentiate between rifles, shotguns, and army firearms—the most we can say is that there was evidence of a decrease in estimated trends in male firearm suicide (in the total sample, and in ages 15–34 years old) after 1990, and in male firearm homicide after 2003, which may be related to two laws (no change or increased rates in the reference group). However, other factors than stricter firearms legislation may be related to the changes in suicide rates in males and that these factors had an overall effect on the occurrence of suicide by all methods. Such factors might include prevention strategies, efforts to improve mental health services, and the chain of care for patients with intentional self-harm. In fact, a general reduction in suicide rates by approximately 25% was observed in Norway from 1988 to 1994, *i.e.*, before the suicide prevention plans were implemented starting in 1994 [27]. From 1994 to 2009, during a period when the prevention plans were active, male non-firearm suicide rates did not change significantly (Table 4, Figure 1). Mental and behavioral disorders, as well as history of intentional self-harm (including suicide attempts), are strong risk factors for suicide [33]; thus, improved mental health care (both inpatient and outpatient hospital care), and follow-up care for patients who have been treated for intentional self-harm, may in all likelihood prevent suicide. After 1990, and especially during the years from 1998–2006, resources in mental health services increased in Norway. However, this change was not significantly related to either male or female suicide mortality [34]. Chain of care for patients who have received emergency medical treatment for intentional self-harm is another factor that may be related to change in suicide rates. However, no differences in changes of suicide rates were observed when Norwegian population areas with and without chain of care intervention were compared [35]. These findings may reflect the complexity and multiplicity in factors that contribute to the occurrence of suicide [36]. Our observational study was unable to separate any possible effect of firearms legislation from the effects of other factors; thus, we cannot rule out that the firearms laws had no effect on firearm suicide among young males.

Other authors have also reported a decrease in firearm suicide among young males after stricter firearm regulations, as we observed after 1990 (shotgun regulation). Gagné *et al*. [11] reported from Quebec, some years after the implementation of Bill C-17 in 1991 to require safe firearm storage, the pace of the decline in firearm suicide rates was twice as great among men aged 15–34 years as among men 35–64 years. Total suicide rates also declined in these two age groups, and Gagné *et al*. [11] conclude that among young men “restricting access to firearms in this age group might be effective in reducing suicides”. There are questions about substitution. A previous study by Caron *et al*. [37] analyzed the impact of the same law, also in Quebec, and found support for the substitution hypothesis: firearm suicide was replaced by hanging among males. McPhedran and Baker [38] tested the impact of a cost-intensive 1996 reform on suicide among young people in Australia; more than 600,000 firearms were bought back from private owners and destroyed by the police [17,39]. The authors found no evidence that the law had any impact on the 15–34 age group, and suggest that “these findings contribute to the growing body of evidence documenting the limitations of various forms of method restriction as a means of addressing youth suicide” [38].

Different hypotheses have been presented regarding firearm restrictions and suicide [3,4,17,36,40]. One hypothesis states that removing or controlling access to means of suicide is an effective prevention strategy at both individual and population levels; this is most likely to be effective when it targets both vulnerable groups and commonly used methods. Another hypothesis states that legislation has more or less no impact, or that alternative methods will be used if restrictions apply to the preferred method (e.g., restricted firearm accessibility), hence the total suicide rate will remain unaffected.

Published literature is inconsistent regarding the effect of means restriction. A review of studies about means restrictions [24] concluded that the risk of substitution with other methods appears to be small; studies tend to indicate that individuals have a preference for a given means, which limits the likelihood of substituting another method. In contrast, other studies have claimed that method substitution does occur [3,37]. De Leo *et al*. [41] concluded that an observed increase in suicide by hanging in Australia, which happened as suicide by firearm decreased, could not be explained by method substitution alone. The authors suggested that the shift from firearms to hanging could be explained by a combination of gun control legislation and changes in the social acceptability of particular methods of committing suicide.

Regarding homicides, we observed a significant impact on firearm homicide risk after the governmental removal of National Guard weapons stored in private homes. It is assumed that this only affected the group impacted by the legislative change. However, there is no data available to explore this assumption. Associated to specific impact on military personal, Lubin *et al*. [12] reported that suicide due to firearms declined significantly after a policy change in the Israeli Defense Forces in 2006, which dictated that soldiers must leave their weapons on base when they go home for weekend leave. There are however, more studies that failed to demonstrate measurable effect of legislation on homicide by firearm, as in a recent study from Canada: This study examined firearm homicides at national level from 1947 to 2008, and relatively few effects of the legislative changes were observed [42].

In contrast to findings from other Nordic countries [15], we did not observe any effect of the 1986 regulation (mandatory examination of hunters) on unintentional firearm deaths. Any potential effect was overridden by a notable and steady reduction in the number of deaths from 1969 on. This reduction goes back to the 1950s [43], when the mortality rate attributed to unintentional firearms and explosive materials was as much as 30–40 times higher than the risk level observed at the end of our study period. Additionally, our failure to discern an effect of the 1986 regulation may be due to the method we used (the period used to measure the effect of the intervention in the piecewise regression only covered data from 1986 to the implementation of the next law in 1990, Figure 4). Moreover, we observed a low incidence of accidental firearm deaths. Also of note, our data set covered all accidental firearm deaths, not only hunting-related fatalities. Studies in the United States and Sweden suggest that between 30% and 50% of all unintentional firearm fatalities are related to hunting [2,15]. More detailed studies of both firearm homicides and accidental firearm deaths in larger countries might assist in evaluating the effects of the laws.

Lack of specificity in the classification system of ICD-10 compared with ICD-9 limits the possibility of obtaining important information about firearm type [44]. In our study, the data available from 1996 was classified by ICD-10. The fact that close to one-half of firearm deaths were coded as caused by “other and unspecified firearm” makes it even harder to determine whether handguns were used less than other types of firearms, as our results suggest. Missing information about firearm type in official mortality data has also been reported in other countries, including the United States [45]. In the United States, another information system for violent and firearm-related deaths is available, and a study of suicides among youths (<18 years of age) showed that the deceased were nearly as likely to use a long gun as a handgun [46].

### Limitations

A number of limitations should be considered when interpreting findings from our study. In public health policy, legal regulation is used as a means to control the environment and improve safety and health, such as reducing fatal firearm injuries [14,20,47]. However, assessing and quantifying a possible impact of a new law is a challenge, e.g., how to measure impact of the legislation for mandatory training for hunters in Norway after 1986. So, the design of our study has limitations, one is that the definition of the period lengths used in the piecewise regression cover very different numbers of years (some a decade, other three years). Further, not all laws have an implementation date of 1 January so some annual figures cover both pre and post legislation. Another limitation is that even if the laws were effective to reduce firearms mortality, each of the laws may have a small impact on the overall number of firearm deaths in the long-term, and a gradual impact over time. This fact combined with the small number of deaths, particularly in the category of accidental firearm deaths, may increase the risk of type II error [48]. However, the caution about sample size is unlikely to apply to firearm suicides, as the sample size for this category was quite large. Another common problem in such “naturalistic experiments” is that we cannot isolate the effect of a single intervention from effect of other factors, and there are probably confounding social factors that affected the pre-legislation and post-legislation periods.

During the last few decades, the increasing focus on safety and injury prevention together with a change in the public’s behavior and attitudes may have reduced fatal firearm injuries. Legal interventions are only one of many factors associated with such a change in safety culture. So, other factors may have played a role, e.g., such as switching the hunter’s goal from providing food to recreation, adventure and safety.

The method we used to measure the impact of the four firearm laws, with a few years between each implemented law, can “hide” effects. Regression coefficients that were not significant are not necessarily unimportant, but may reflect small sample sizes. Regarding quality of data, misclassification of the external cause of injury may be a problem (e.g., for unintentional firearm deaths) [49]. As classification of the external cause of firearm deaths may have changed over time, we included all firearm deaths in this study, independent of intent. However, a number of firearm injury deaths may be missing because of an increased number of deaths registered without cause-of-death information since the late 1990s [27]. Additionally, in other countries such as Australia, there is concern about the deteriorating quality of official suicide mortality statistics [50,51,52,53,54], and one consequence is that the impact of firearm laws in public health strategies must be re-evaluated based on more reliable data [55,56].

## 5. Conclusions

European studies of the impact of firearm laws are still rare. This study from Norway demonstrated evidence that two of the four laws could have contributed to the decreased risk in male firearm mortality. However, the data showed also inconclusive results, which might be a result of limitations in the design and the data itself, or may reflect that the firearm laws under study, had no effect. Thus, additional research is needed to answer what (if any) impact stricter firearms laws had on firearm deaths in Norway. However, the present study adds to the growing literature regarding the impact of firearm control on firearm deaths and injury prevention.
